# Individual Responses of Captive Amazon Parrots to Routine Handling Can Reflect Their Temperament

**DOI:** 10.3390/ani13040738

**Published:** 2023-02-18

**Authors:** Gabriela Ramos, Victor Araújo Franzone Vital, Talys Henrique Assumpção Jardim, Gustavo Nunes, Maria Eduarda Caçador Branco, Cristiano Schetini de Azevedo, Aline Cristina Sant’Anna

**Affiliations:** 1 Programa de Pós-Graduação em Biodiversidade e Conservação da Natureza, Universidade Federal de Juiz de Fora, Juiz de Fora 36.036-900, Brazil; 2 Núcleo de Estudos em Etologia e Bem-Estar Animal, Departamento de Zoologia, Universidade Federal de Juiz de Fora, Juiz de Fora 36.036-900, Brazil; 3 Programa de Pós-Graduação em Ciências Veterinárias, Universidade Federal Rural do Rio de Janeiro, Seropédica 23.890-000, Brazil; 4 Departamento de Biodiversidade, Evolução e Meio Ambiente, Universidade Federal de Ouro Preto, Ouro Preto 35.400-000, Brazil; 5 National Council for Scientific and Technological Development, CNPq Researcher, Brasília 71.605-170, Brazil

**Keywords:** ex situ conservation, manual restraint, personality

## Abstract

**Simple Summary:**

Temperament tests, manual restraint, and behavioral training were applied to investigate the relationship between temperament and response to manual restraint and evaluate whether parrots exhibited higher responsiveness to physical restraint after training to increase flight capacity and human aversion. This study discusses the relationship between the temperament trait of fear and parrots’ responses to manual restraint test and explores factors which may have influenced these responses. It is suggested that physical restraint during routine procedures in captivity, such as blood collection, can be an option to assess individual behavioral differences related to fear and individual responses to stressful stimuli in parrots. However, it is not possible yet to dismiss temperament evaluations through behavioral tests, to ensure a broader analysis of temperament.

**Abstract:**

Individual responses to physical restraint and temperament have been assessed in birds of several species; however, there is a paucity of research which investigates both aspects, especially in captive parrots. This lack of studies raises doubts about which temperament traits, if any, are evidenced during handling and if the intensity of responses to restraint is affected by behavioral training programs, a common practice used in ex situ conservation programs. To understand more about the subject, this study aimed to identify the main temperament dimensions of parrots and investigate their relationship with response to physical restraint for blood collection. A secondary aim was to evaluate whether parrots exhibited higher responsiveness to physical restraint after training to improve flight capacity and increase aversion to humans. The main dimensions identified were activity, neophilia, vigilance, and fearfulness. The more fearful parrots in temperament evaluations were more responsive to physical restraint, showing more vocalizations and struggle attempts than the less fearful ones. After training, the parrots showed higher responsiveness to physical restraint. We suggest that physical restraint for routine handling, such as blood collection, could be a feasible option for centers of rehabilitation to use to obtain data on individual behavioral differences in fear responses.

## 1. Introduction

Temperament, personality, or coping styles are concepts used to explain individual behavioral variations in non-human animals that persist over time and in different situations, indicating that animals from the same species, age, and sex can show their individuality and do not behave in the same way [1,2,3]. This individuality can be represented by dimensions/temperament traits that can predict certain behavioral responses of animals, for example, alternative response patterns in reaction to a potential stressor [4].

Temperament dimensions can be assessed through standardized tests, in which new or challenging situations and new stimuli are presented to the animal individually (i.e., a novel object, an unknown person, or a novel environment) [2,5]. In this case, behaviors expressed are recorded quantitatively or qualitatively. The observers can assign scores to the animals in adjectives pre-selected by the researchers (qualitative assessment [6]) or they can record the duration or frequency of behaviors such as vocalizations, body movements, exploration, resting, and alertness after the presentation of the stimulus for a predetermined time (quantitative assessment [7]). Novel object, novel environment, and open field tests are evaluations widely used to evaluate individual differences in several species [1,7,8,9]. These tests expose the animals to novelty and/or threat stimuli, thereby revealing dimensions of boldness, neophobia, fearfulness, exploration, and activity [2].

The recording of behavioral and physiological responses to physical restraint can also be used to assess the animals’ individual responses to fear and stress (stressful and fearful situations) [10]. This type of evaluation has been used for bird and mammal species and is reported in the literature with a range of different names and varying types of restraint (e.g., manual restraint, handling tests, physical restraint tests, and struggle rates). Examples include manual restraint tests in zebra finches (*Taeniopygia guttata* [11]), laying hens (*Gallus gallus* [12]), parrots (*Psittacus erithacus* [13]), and cockatiels (*Nymphicus hollandicus* [14]); handling test or handling stress in great tits (*Parus major* [15,16]) and laying hens [17]); physical restraint tests in laying hens [10]; and struggle tests or struggle rate in zebra finches [18], squirrels [19], and domestic cats [20]. When submitted to physical restraint, animals can react actively or passively (passive or active avoidance) [10,11]. These two coping styles can also be related to physiological differences in response to stressful stimuli [4]. 

In laying hens [10,21] and passerines [15,16], passive and active avoidance are two possible behavioral strategies in response to fearful or stressful situations, for example, when under threat of predation or physical restraint [10,22]. Passive avoidance occurs when the bird shows less physical resistance to handling by humans or to the human approach test, remains still (freezing), or has a higher latency to fly after a longstanding restraint (showing tonic immobility) in evaluations such as handling tests, tonic immobility tests, struggle tests, and physical restraint tests [10,15,18]. On the other hand, active avoidance occurs when the bird reacts to stressful situations more intensely, with a higher frequency of wing flaps (more resistance to handling) and a lower latency to fly after restraint, not showing tonic immobility [10,15,18] but characteristics of the fight or flight response. 

Studies that use the manual restraint test in parrots usually seek to obtain physiological and behavioral parameters of the stress response to the restraint [23,24,25,26]. A significant increase in the cloacal temperature after 4 min of manual restraint and an increase in mean respiratory rate during a 15-min period of manual restraint was observed in *Amazona aestiva* and *A. ventralis* [27]. After going through a stressful event (manual restraint and physical examination), cockatiel (*Nymphicus hollandicus*) had increased concentrations of circulating corticosterone and showed behavioral changes, including more resting and reduced locomotion, feeding, and interaction with the environment [14]. To our knowledge, only one study has used physical restraint to assess the temperament or personality of parrots through evaluation of individual responses to the restraint stress [13]. If routine handling that involves physical restraint could be used to assess individual variations to fear and stress, just like the manual restraint test, it would be possible to use routine handling to investigate the parrots’ temperament. 

The genus *Amazona*, just like other neotropical parrots, is under threat due to the culture of keeping them as pets and increasing deforestation [28]. Parrots are frequently found in rehabilitation centers, zoos, breeding centers, and ex situ conservation programs where individuals may be released into the wild. Knowing the individual responses to humans, novelty, and stressful situations and incorporating these into behavioral profiles is important in developing welfare-friendly handling, which minimizes stress for birds, considering each animal’s profile and how they deal with these situations.

Meanwhile, behaviors expressed during restraint, characterized as “responsiveness to handling”, can be desirable for animals that are going to be released. More intense responses to human restraint (e.g., more pecks at the handler, more vocalizations, and more struggle attempts) can decrease their chances of being captured after being released in the wild. If individual behavioral responses to routine handling express the variations of avoidance strategies (active/passive avoidance) in the group, they could be useful to predict animal temperaments, and traditional behavioral tests could be dismissed. These tests involve procedures that are more sophisticated and more difficult to be executed by the institutions that protect wild animals (rehabilitation centers, zoos, breeding centers, sanctuaries, and wildlife maintainers). Thus, it is beneficial to evaluate if parrots’ response to routine blood collection handling is correlated with their behavioral responses in tests traditionally used to assess temperament. It is also important to investigate if the intensity of the behavioral response to restraint handling changes after the animals have been through pre-release rehabilitation procedures. Such training is frequently used in release programs, and it can change individual behavioral patterns [29].

We tested the following predictions: (a) animals that are less responsive to restraint handling (animals that show a lower frequency of wing flaps, fewer vocalizations, and fewer attempts to peck the handler) will be more shy in the tests of novel objects and reactions to humans (remaining far from the stimuli, with more caution); and (b) the intensity of the parrots’ responses to handling will increase after going through a pre-release rehabilitation process, which involves improvement of physical conditioning for flight and human aversion training.

The objectives of the present study were: (a) to relate the temperament of Amazon parrots with their behavioral responses to physical restraint for blood collection; and (b) to investigate whether their behavioral responses to physical restraint are consistent before and after flight training and human aversion training.

## 2. Materials and Methods

### 2.1. Animals and Study Site

Thirty-eight parrots were evaluated: blue-fronted Amazon parrot (*A. aestiva*, *n* = 23), vinaceous-breasted Amazon parrot (*A. vinacea*, *n* = 10), and red-browed Amazon parrot (*A. rhodocorytha*, *n* = 5). These parrots came from the Wild Animals Triage Center (CETAS) of Juiz de Fora. The animals were transferred to a Wild Animal Release Area, where they were kept from March 2021 until November 2021, a period referred to as acclimatization or pre-release rehabilitation. Flight training and human aversion training were performed as part of the pre-release rehabilitation. Parrots were tested monthly to assess the ongoing effectiveness of the training. Additionally, temperament tests, blood and feces collection for parasitological exams, sexing, behavioral evaluations from direct observations, and body weight and plumage condition recordings were conducted.

A flight and human aversion training protocol was applied to the animals for ten weeks. The flight conditioning was used to improve the parrots’ flight capacity and it was executed four times per week, in the afternoon. In these training sessions, the animals were stimulated to fly by using a capture net to simulate a capture for 5 min. Human aversion conditioning was used to decrease the positive relationship with humans, and it was executed three times per week, in the morning. In these training sessions, an evaluator offered each parrot sunflower seeds, and if the parrot accepted the food, the evaluator would shake a can filled with stones, making a high and disturbing noise to the animal. More details are available in [30].

During the pre-release acclimatization, the parrots were kept in two aviaries, one with 13 animals (8.50 m length × 4.60 m width × 3.50 m height), and another with 25 animals (10.50 length × 7.30 width × 3.14 height). In both, the animals were fed twice a day (morning and afternoon) with extruded ration, sunflower seeds, fruits, and vegetables (apple, banana, papaya, mango, jabuticaba, orange, chayote). Water was available through a natural source ad libitum.

### 2.2. Temperament Tests

Two temperament tests were applied to the animals, individually and sequentially: novel object test (NO) and reaction-to-unknown-person test (RUP). These two tests are usually conducted for bird personality evaluations [7,18,31]. Both tests were executed three times per animal. The interval between the first and the second test was 15 days and the interval between the second and the third test was 127 days.

**Figure 1:**
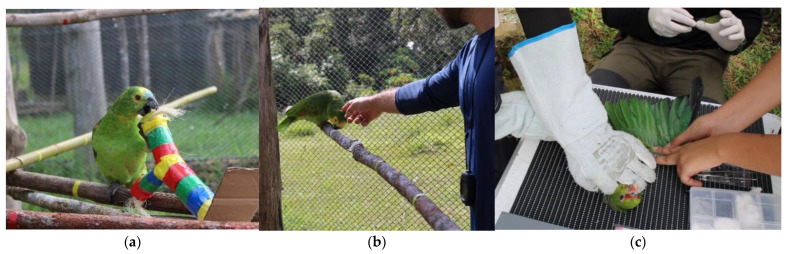
(**a**) Interaction between the parrot and the colored stick used as a novel object in the first novel object test; (**b**) flight distance measurement performed after the unknown person reaction test; (**c**) manual restraint test: blood collection for parasitological exams in which behavioral variables were recorded during physical restraint.

In each test, a parrot was captured randomly, put inside a cage covered with a black cloth (35 cm × 42 cm × 45 cm), and transferred to the aviary in which the tests were conducted (8.50 m length × 4.60 m width × 3.50 m height). The cage was positioned on a pedestal at the center of the aviary with the door open. The parrot remained in the cage until they came out into the aviary willingly. Once they were out in the aviary, they were given a 5-min habituation period before the novel object was added to the aviary. The same habituation time was used in the RUP test. The tests are described below:

- NO: After the habituation period, a novel object was added into the aviary, and the behaviors were recorded for 5 min. A toy train, a colored stick (yellow, blue, red, and green), and a yellow hat with colored dots were used as novel objects in the first, second, and third tests, respectively (Figure 1A).
- RUP: After removing the novel object and waiting for the habituation period, an evaluator assumed a position in the aviary and remained still for 5 min, while a second evaluator recorded the birds’ behavioral responses. After that, the evaluator that was still approached the parrot with their hand outstretched. Flight distance and the latency to react to the approach were recorded (Figure 1B).

Behaviors were recorded using focal sampling with instantaneous recording with 20-s intervals. The behavioral categories observed were as follows: time away (≥2 m) from the novel object and the person, alertness, inactivity, preening, locomotion, environment exploration, novel object exploration (recorded in % of the observation time), vocalization (number of occurrences), latency to touch the object (in seconds), novel object touch (number of occurrences), and flight distance in the reaction-to-unknown-person test (in centimeters) following Ramos et al. [32]. Full descriptions of the behaviors recorded are included as Supplementary Material (Table S1). 

### 2.3. Manual Restraint Test

The physical restraint of the parrots during blood collections for parasitological exams was performed twice during the pre-release acclimatization period and will be referred to as the “manual restraint test”. The birds’ responses to the manual restraint test were used to assess individual behavioral responses to physical restraint during a routine exam of the parrots, and to determine the birds’ responses to handling. The assessment of individual responses to physical restraint or human approach can be found in the literature for other bird species, such as laying hens [10,21], parrots [13,25,26,33], and passerines [15,16,18]. The animals in the studies cited above underwent physical restraint, but there was no blood or biological material collected, as we did. In the present study, the behavioral variables of the manual restraint test were recorded during the physical restraint for the blood collection.

The blood collection was carried out through a brachial vein puncture in one of the wings. The volume of blood collected did not exceed 0.5 mL or 1% of the total weight of the animal [34,35]. The puncture was carried out with a sterilized needle (0.45 mm × 13 mm—subcutaneous pattern) attached to a 1 ml syringe with the bevel facing up.

Tests were filmed for future analyses and undertaken by only one previously trained evaluator. From the moment the animal was positioned on the table for the exam, the following behavioral variables were monitored: handling duration (in seconds), struggle/escape attempts, vocalization, and pecks at the handler (in number of occurrences) (Table 1, Figure 1C). The test was finished when the evaluator responsible for the blood extraction had finished the sample collection procedure. The manual restraint test was executed twice with the parrots, before and after the flight and human aversion training. Different people handled the animals in the two evaluations; however, the way to position the hands and hold the animal on the table were standardized and kept the same. The blood collection and the behavioral recordings were completed by the same evaluators.

### 2.4. Statistical Analyses

Descriptive statistics were calculated for each variable and a Kolmogorov–Smirnov test was used to assess normality.

Factor analysis was applied to the variables obtained from both temperament tests (time away _NO_, time away _RUP_, alertness _NO_, alertness _RUP_, inactivity _NO_, inactivity _RUP_, preening _NO_, preening _RUP_, locomotion _NO_, locomotion _RUP_, environment exploration _NO_, environment exploration _RUP_, vocalization _NO_, vocalization _RUP_, novel object exploration, novel object touch, latency to touch the novel object, latency to react to unknown person’s approach, and flight distance) to reduce the data dimensionality and to obtain the main dimensions of parrots’ temperament [36]. Following confirmation of consistency in responses across the replicates (*p* > 0.05), the mean of the three repetitions of the tests for each behavioral variable was used for factor analysis. The first four factors with eigenvalues above 1.0 were retained, and variables with loadings ≥ 0.50 were considered as main contributors to the dimensions (factors). Animal scores in the dimensions were regarded as the individual’s temperament.

Mixed linear models and generalized linear mixed models (GLMM) with the use of PROC MIXED and PROC GLIMMIX (SAS Inst. Inc., Cary, NC, USA) were applied to assess whether the parrots were consistent between the three temperament evaluations and the two restraint evaluations and to assess the effect of sex, species, and their interaction (sex*species) on individual temperament and the variables from the restraint test. The models included the main temperament dimensions and each variable observed in the manual restraint test as dependent variables. Factor 2 was included in the models as a dependent variable with lognormal distribution (*logn*). The fixed effects of species, sex, and evaluation (three repetitions for temperament and two for the manual restraint test) were considered. Animal (*subject*) was added as a random effect to control for repeated measures throughout the evaluations. 

Spearman’s rank correlation was used to assess the relationship between the variables obtained from the manual restraint and the temperament tests. Significant correlations (*p* ≤ 0.05) were retained. Graphs were created with the *ggplot2* package via RStudio [37].

## 3. Results

### 3.1. Temperament

Parrots were consistent in their responses across the three repetitions of the temperament tests since no differences were found across the evaluations (*p* > 0.05 for the four dimensions). The first four factors from the factor analysis were retained to obtain the main temperament dimensions: activity, neophilia, vigilance, and fearfulness (Table 2). Together, the four factors explained 58.75% of the variation in the dataset.

The first factor, activity, explained 22.04% of the data variance. The variables with high positive loadings for this dimension characterized active animals (vocalization _NO_, vocalization _RUP_, locomotion _NO_, locomotion _RUP_, and environment exploration _RUP_) and the variables with high negative loadings for this dimension characterized inactive animals (inactivity _NO_, inactivity _RUP_). Animals with higher scores for this factor were considered to be more active.

The second factor explained 16.61% of the variance in the dataset and was interpreted as neophilia. The variables with high positive loadings that characterized this dimension were novel object exploration and novel object touch. Parrots considered neophiliac interacted for a longer time and touched the novel object more times than those considered to be more neophobic, which did not show (or showed less) those behaviors. Higher scores in this factor indicated more neophiliac animals.

The third factor explained 10.30% of the variance and was identified as vigilance. The variables with high positive loadings for this dimension (environment exploration _NO_ and inactivity _RUP_) characterized the indifferent parrots, and the variables with high negative loadings (alertness _NO_, alertness _RUP_) the vigilant ones, which remained alert to the stimuli, unknown person, and novel object. The more vigilant animals were those with the lowest scores in factor 3.

The fourth factor was interpreted as fearfulness and explained 9.80% of the variance in the dataset. Only variables with negative loadings were obtained in this factor, such as time away _NO_, time away _RUP_, and flight distance. Thus, the most fearful parrots (i.e., the ones with lower scores in this factor), were the ones that remained more distant from the novel object and the person during the tests (time away _NO_, time away _RUP_) and showed a greater flight distance (i.e., did not allow approach). Less fearful parrots, with higher scores in this factor, remained distant from the stimuli for less time and showed a shorter flight distance (allowing the observer to approach).

Investigation of the impact of the effect of sex, species, and interaction (sex*species) on the four temperament dimensions showed a significant effect only for the third factor (vigilance). *A. aestiva* parrots (mean ± SD; 0.30 ± 1.02) were less vigilant than *A. vinacea* (−0.49 ± 0.92) and *A. rhodocorytha* (−0.41 ± 0.50, F_2_ = 3.64; *p* = 0.04). There were no other significant differences. 

### 3.2. Responses to Manual Restraint 

There was a significant change in behavioral responses to the manual restraint test before and after the training protocol. After the flight and human aversion training, the parrots showed more struggle attempts (F_1,72_ = 4.38; *p* = 0.03), more vocalizations (F_1,72_ = 7.53; *p* = 0.008), and longer handling durations (F_1,72_ = 28.14; *p* < 0.0001) (Table 3). 

There was a significant effect of the interaction sex*species for vocalization in the manual restraint test (F_2_ = 3.27; *p* = 0.05). Females vocalized more than males in *A. vinacea*, and for *A. aestiva* and *A. rhodocorytha* they did not differ (Table 4).

### 3.3. Relationships between Responses to Manual Restraint and Temperament Tests

**Figure 2:**
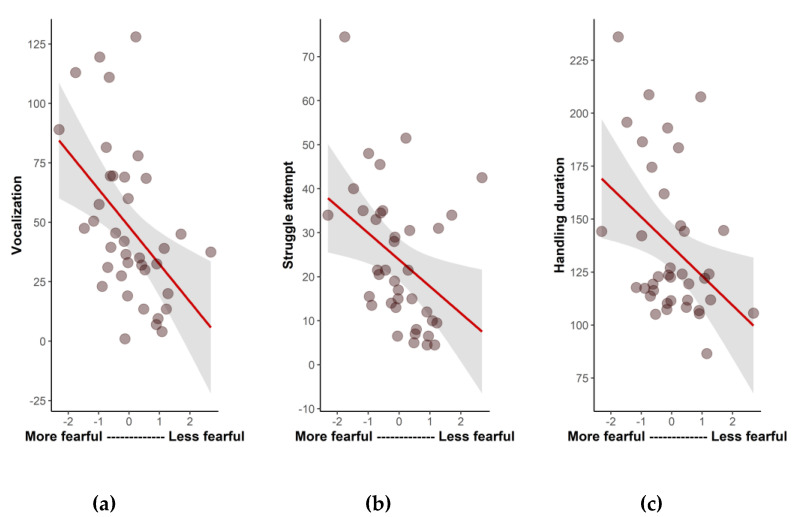
Significant Spearman’s correlations between the fearfulness temperament dimension and the variables obtained during the manual restraint test) for the 38 parrots: (**a**) vocalization; (**b**) struggle attempts; (**c**) handling duration (s).

There were significant correlations between the fearfulness dimension and three variables from the manual restraint test, indicating that more fearful animals (i.e., with lower scores in this dimension) showed more struggle attempts (r_s_ = −0.49; *p* = 0.002), more vocalizations (r_s_ = −0.52; *p* = 0.008), and had a longer handling time (r_s_ = −0.35; *p* = 0.03) during the manual restraint tests than less fearful parrots (Figure 2). The other three temperament dimensions (activity, neophilia, and vigilance) were not correlated with the variables of the struggle test (*p* > 0.05).

## 4. Discussion

In this study, parrots displayed diverse behavioral responses to the stimuli (person and novel object) and to the restraint test. Four main temperament dimensions were identified as activity, neophilia, vigilance, and fearfulness. Parrots were consistent between the three repetitions of the temperament tests, even after the training protocol. With respect to the response to physical restraint, parrots showed more intense responses after the flight and human aversion training protocols. Responses elicited from the physical restraint for blood collection correlated with the dimension fearfulness, reflecting this component of the parrots’ temperament in the restraint test. Knowing how these stable individual differences in behavior [38,39,40] are expressed during routine handling could be an important tool to evaluate the parrots’ temperament, which could then be used to improve husbandry. 

The first factor extracted from the temperament evaluation was interpreted as activity. Active animals moved more, explored the environment more, and vocalized more in the temperament tests, showing more agitation and exploratory behavior when presented with the stimuli. Inactive parrots spent more time resting during the tests. The behaviors that characterized this profile (inactivity _NO_, inactivity _RUP_) were recorded when the animals were still, relaxed, and often with their eyes closed, even in the presence of stimuli (the person and novel object). Individual differences in the patterns of activity and movement of the animals were identified by standardized tests, such as the open-field test, the novel environment test, or the cage activity test in previous studies, enabling the classification of the birds into two coping strategies (higher/lower level of general activity) [2,7,13,18,41].

The second factor was interpreted as neophilia, reflecting parrots that explored and touched the novel object more. Neophilia describes the animals’ levels of exploration, in which investigation and interaction are motivated by a novelty in the environment, represented in this study by the novel object [42]. Animals with high levels of neophilia, which are curious and more prone to interact with novelty, can show a tendency to explore more sources of food, partners, nesting places, and shelter; however, they can show less caution in the presence of humans or new and potentially dangerous situations [5]. High levels of neophilia or the loss/decrease in neophobia (possible consequences of captivity [31,43]) can have implications for the conservation of these individuals since neophiliac individuals should be more prone to risks [44]. For this reason, it is important for institutions that keep parrots in captivity to consider individual responses to novelty, especially where there are plans for releasing these animals into the wild.

The vigilance dimension is less explored in the literature compared to activity and neophilia. There is evidence of individual variation in vigilance in wild and captive birds [45,46,47], including for other parrot species [7,48]. In nature, these animals would face a trade-off between being alert to external stimuli to decrease the risk of predation and engaging in other routine activities (e.g., feeding). Even though antipredatory behavior can be weakened in captivity because the selective pressure of predation is absent, the findings of the present study reinforce the presence of individual- and species-level variations in this trait in captive birds. *A. aestiva* parrots were less vigilant than *A. vinacea* and *A. rhodocorytha* parrots. However, there were variations in number of birds of the three species and so this species-level difference should be interpreted with caution.

Fear can be defined as a complex emotional state induced by danger, threat, or novelty. It is related to physiological processes that are activated to protect animals from situations that can threaten their development, reproduction, and survival [22,49,50]. In this study, factor 4 had higher loadings for distances from the stimuli, being interpreted as representing the fearfulness dimension. Thus, our study suggests the existence of individual variations of fear in response to both the unknown person and the novel object.

Studies that have used manual restraint on captive parrots are still few in number [13,14,23,24,25,26,27] and, generally, the studies investigated physiological responses to restraint. Physiological changes following restraint include increased levels of corticosterone [14] and increases in cloacal temperature and respiratory frequency [27]. For example, Van Zeeland and colleagues [13] applied the manual restraint test and two temperament tests (open field and novel object) to grey parrots (*Psittacus erithacus*) to obtain the proactive–reactive axis for each test separately. Relating to struggle parameters, proactive parrots in the novel object and open field tests had high levels of feather damaging behavior, vocalized less and for less time, and tended to fight more against the handler in the manual restraint test [13]. No significant correlation was observed between the type of coping in the manual restraint test (proactive/reactive) and the presence of feather pecking behavior. Unlike the present study (in which the variables of all temperament tests were combined in a single factor analysis), these authors extracted the dimensions for each test separately; additionally, Van Zeeland et al. [13] did not assess the relationships between the temperament tests (novel object and open field) with the variables from the restraint test, as we did. 

In our study, more fearful parrots in the temperament tests vocalized more, struggled more, and had longer handling durations during physical restraint. It was expected that more fearful, shier, and more risk-averse parrots would react less intensely to the physical restraint (passive avoidance). This hypothesis was formulated according to studies that applied physical restraint to extract response profiles to handling stress in passerines (wild-caught hand-reared birds) [1,15,18], captive parrots [13] and laying hens [10,12,21,51]. These studies suggest that birds regarded as having “slow explorer”, “shy”, “reactive”, or “low feather pecking” profiles show lower levels of activity and aggression and higher levels of immobility, reflecting the passive response pattern to handling stress. Interestingly, the contrary happened in this study. More fearful parrots responded to the physical restraint by adopting an active avoidance strategy in response to stress, vocalizing more, struggling more, and requiring longer handling times (due to the difficulty of containing them). The more fearful/shy profile showed active avoidance responses to fear and stress probably because they understood the physical restraint as an annoying and aversive stimulus since this profile was characterized in the temperament tests by remaining distant from both the person and the novel object during assessments. 

More fearful parrots also showed more vocalizations than the less fearful ones. In van Zeeland and colleagues’ study [13], grey parrots without feather pecking behavior (reactive to open field and novel object test) vocalized more and for longer times than the ones with feather pecking behavior (proactive) in the manual restraint test, a trend that arose similarly in laying hens [21]. The authors suggest that parrots can vocalize louder and longer in situations of fear and stress to alert other members of the group about predators, allowing escape and survival. Thus, more fearful animals (reactive, without feather pecking behavior) showed more vocalizations [13]. This response was observed in the present study and a similar reason is suggested here. 

The parrots showed more intense responses to physical restraint after the training periods. Both types of training (flight and human aversion) had stimuli considered partially aversive or annoying to the parrots in addition to having been applied by humans, a fact that may have influenced the parrots’ responses in the second restraint test repetition, making the animals more reactive to handling. It is important to highlight that, before the training protocol, 57.89% of the parrots accepted the food from the person’s hand, and after the training protocol, 81.57% not only rejected the food but also moved away from the person, as reported in a previous study from our research group with these individuals [30]. During the present study, the contact with humans was restricted to the behavioral evaluations and to the feeding, in which the handlers would wear a camouflage cloth that disguised the human silhouette to feed the parrots. Thus, it is possible to assume that a pre-release acclimatization period with flight and human aversion training can influence individual responses to physical restraint, making them more intense.

Even though previous studies assessed individual differences in the propensity to risk and aspects of response to humans with the restraint test, analyzing these factors separately or together, we did not find studies that correlated individual responses to restraint to the main temperament dimensions in captive Amazon parrots. Thus, the present study is an important starting point for more accessible temperament evaluations in places such as rehabilitation centers for individuals from this group. Our study had some limitations that must be acknowledged. First, the restraint test repetitions should ideally have been done by the same researcher, but it was not possible due to logistical reasons. However, both researchers were experienced and were trained to restrain wild parrots in the same place. The restraint position was also standardized for consistency, being the same in the two evaluations performed. A second limitation was the difference in the sample sizes for the species studied, which means the reported species-level differences should be interpreted with caution.

## 5. Conclusions

In this study, we aimed to assess individual behavioral differences using temperament tests that are widely used in the literature for several captive parrot species and relate them to parrot responses to physical restraint. A further aim was to evaluate whether behavioral responses to handling similar to the ones used in centers of rehabilitation and zoos (i.e., restraint tests) could reflect components of parrot temperament, and in doing so, propose a more accessible option for these institutions to use to obtain data on individual behavioral differences in captivity. Our results showed that there was a correlation between fearfulness and most of the behavioral variables observed (level of struggle, vocalizations, and handling times in the manual restraint tests), with more fearful parrots showing an active coping strategy in response to the stress. We showed that responses to physical restraint in routine handling can express variations of fear and can be useful to predict this aspect of temperament in captive parrots. However, the other dimensions, which are also relevant for birds that will be released, such as neophilia and vigilance, cannot be obtained with the restraint test. This reveals that it is not possible yet to dismiss temperament evaluations through behavioral tests, to ensure a broader and more comprehensive analysis of these characteristics.

It is important to know how individual animals may react to changes in the physical or social environment (such as environmental enrichments and changes in the group’s social dynamics) and stressful stimuli such as physical restraint. Such contexts occur frequently in rehabilitation centers, zoos, reproduction centers, and ex situ conservation programs. Being able to predict individual behavioral responses can be useful in altering environments and handling conditions according to the animal’s individual temperament differences.

Finally, we noted that reactivity to physical restraint can be intensified after a training protocol, which is generally used in reintroduction programs for parrots to improve behavioral abilities essential to their survival in the wild. This outcome is favorable when these animals are destined to be released.

## Figures and Tables

**Table 1:** Behavioral categories (response variables) recorded during the manual restraint test.

Behaviors	Description
Vocalization	Any sound emitted by the bird during the restraining for the blood collection.
Peck at the handler	When the bird opens and closes its beak, pressing the handler’s hand, pecking.
Struggle attempt	When the bird flaps its wings and presses its body against the table on which it is laid, trying to avoid physical restraint (they can happen simultaneously or independently). The wing flaps can be shorter or longer (the bird can move both wings backward, making it difficult to position the bird on the table).
Handling duration	It is quantified from the moment the bird is positioned on the table until the end of the blood collection.

**Table 2:** Result of factor analysis for novel object test and the reaction-to-unknown-person test applied to 38 parrots (*A. aestiva*, *A. vinacea*, and *A. rhodocorytha*). The variables retained for the main dimensions are in bold: factor 1—activity, factor 2—neophilia, factor 3—vigilance, and factor 4—fearfulness.

Variables/ Dimensions	Activity	Neophilia	Vigilance	Fearfulness
Time away _NO_^1^	0.07	−0.40	−0.03	**−0.78**
Inactivity _NO_	**−0.50**	−0.38	0.27	0.21
Alertness _NO_	0.02	−0.40	**−0.77**	−0.11
Locomotion _NO_	**0.61**	0.15	0.12	−0.20
Preening _NO_	−0.13	0.23	0.06	−0.08
Environment exploration _NO_	0.37	0.20	**0.67**	0.01
Novel object exploration _NO_	−0.05	**0.94**	0.09	0.09
Latency to touch object _NO_	0.20	0.30	0.04	0.08
Novel object touch _NO_	−0.01	**0.96**	0.07	0.05
Vocalization _NO_	**0.86**	−0.14	−0.05	0.10
Time away _RUP_^2^	−0.02	0.03	−0.06	**−0.87**
Inactivity _RUP_	**−0.66**	−0.13	**0.62**	0.16
Alertness _RUP_	0.28	0.05	**−0.80**	−0.07
Locomotion _RUP_	**0.70**	0.01	0.00	−0.43
Preening _RUP_	−0.05	0.05	0.05	0.09
Environment exploration _RUP_	**0.51**	0.26	0.18	0.19
Vocalization _RUP_	**0.81**	−0.15	−0.12	−0.04
Flight distance _RUP_	0.16	0.00	−0.18	**−0.77**
Latency to react to approach _RUP_	−0.03	−0.12	−0.14	0.06
Eigenvalue	4.19	3.16	1.96	1.96
Variance (%)	22.04	16.61	10.30	9.80

^1^ NO: novel object test; ^2^ RUP: reaction-to-unknown-person test.

**Table 3:** Effect of evaluation (before and after the flight and human aversion training protocol) on the variables of the manual restraint test (estimated means ± standard error).

Repetitions/Variables	Struggle Attempts	Vocalization	Handling Duration (s)	Pecks at the Handler
Manual restraint test 1	19.34 ± 3.05 ^a^	35.95 ± 6.27 ^a^	105.95 ± 8.3 ^a^	37.85 ± 6.24 ^a^
Manual restraint test 2	28.37 ± 3.05 ^b^	60.27 ± 6.27 ^b^	168.29 ± 8.3 ^b^	49.28 ± 6.24 ^a^

Different superscript letters indicate statistical differences between the variables.

**Table 4:** Effects of the interaction sex*species on the number of vocalizations during the restraint test (estimated means ± standard error).

Species	Sex	*n*	Vocalization
*Amazona aestiva*	female	9	32.28 ± 16.70 ^c^
*Amazona aestiva*	male	14	45.75 ± 29.00 ^bc^
*Amazona rhodocorytha*	female	2	64.25 ± 19.44 ^abc^
*Amazona rhodocorytha*	male	3	76.33 ± 37.51 ^ab^
*Amazona vinacea*	female	4	81.00 ± 55.67 ^a^
*Amazona vinacea*	male	6	36.00 ± 27.00 ^bc^

Different superscript letters indicate statistical differences between the variables.

## Data Availability

The data presented in this study are available on request from the corresponding author.

## Supplementary Materials

The following supporting information can be downloaded at: https://www.mdpi.com/article/10.3390/ani13040738/s1, Table S1: Ethogram of the behaviors analyzed during the temperament tests for the Amazon parrots as described by Ramos et al. [32].
