# Supplementary material for: Individual Responses of Captive Amazon Parrots to Routine Handling Can Reflect Their Temperament
Source: Animals (Basel). 2023 Feb 18;13(4):738. doi: 10.3390/ani13040738 (PMC9952057; doi:10.3390/ani13040738)
Supplement: Supplementary file 1 [file animals-13-00738-s001.zip › animals-2222401-supplementary.pdf]

Table S1. Ethogram of the behaviors analyzed during the temperament tests for the Amazon parrots as described by Ramos et al. [32].

**Behavioral category (variable recorded)**	**Description**
Inactivity (duration, in s)	The birds were not doing any of the other described acts and / or sleeping.
Alertness (duration, in s)	Parrots with necks stretched and eyes wide open, focusing on something.
Time away from the novel object and the person (duration, in s)	Time that the bird remained at a 2-m distance from the person, novel object, or model of predator during the whole test.
Flight distance (in cm)	Distance that the bird allowed the unknown person to approach before expressing the first withdrawal response.
Novel object touch (number of occurrences)	Events (in number) in which birds had physical contact with the novel object by using its beak or legs.
Latency to touch the object (in seconds)	Time interval from the moment that the test began until birds had the first physical contact with the novel object by using its beak or legs.
Novel object exploration (duration, in s)	Time that the bird remained in physical contact with the novel object by using its beak or legs.
Locomotion (duration, in s)	Moving sideways or frontally; walking on the ground or on the perches; climbing the mesh or the ceiling; or when it flies.
Preening (duration, in s)	The beak was used for the cleaning of the feathers, the individual approached the beak to some part of the body and pulls the feather, smoothing it; or when scratching, with the head turned to the side and with one of the legs up, using the two anterior digits; taking a shower in the water trough; stretching (in resting position the bird stretches one of the legs or the wings); yawning (still, the bird opens and closes the beak).
Vocalization (occurrences)	Any type of sound emission by the animal when it vocalizes or sings; except human vocalizations.
Environment exploration (duration, in s)	Pecking or handling perches, mesh, sheets, or any object inside the area.
Abnormal behaviors (frequency, occurrences / min)	Repetitive behaviors (stereotypies); bird repetitively pecking at the mesh or walking from one side to the other (Pacing); bird pulling off its feathers.
Imitation of human sounds (frequency, occurrences / min)	The birds vocalized by emitting sounds that imitate human speech (human whistle or words).
